# A Comparative Study of Time Frequency Representation Techniques for Freeze of Gait Detection and Prediction

**DOI:** 10.3390/s21196446

**Published:** 2021-09-27

**Authors:** Tahjid Ashfaque Mostafa, Sara Soltaninejad, Tara L. McIsaac, Irene Cheng

**Affiliations:** 1 Multimedia Research Center, Department of Computing Science, University of Alberta, Edmonton, AB T6G 2E8, Canada; soltanin@ualberta.ca; 2 Arizona School of Health Sciences, A.T. Still University, 5850 E. Still Circle, Mesa, AZ 85206, USA; tmcisaac@atsu.edu; 3 School of Pharmacy and Health Professions, Creighton University Health Sciences, 3100 N. Central Ave., Phoenix, AZ 85013, USA

**Keywords:** Parkinson’s disease, freeze of gait, deep learning, ensemble learning, wearable sensor data, detection and predication

## Abstract

Freezing of Gait (FOG) is an impairment that affects the majority of patients in the advanced stages of Parkinson’s Disease (PD). FOG can lead to sudden falls and injuries, negatively impacting the quality of life for the patients and their families. Rhythmic Auditory Stimulation (RAS) can be used to help patients recover from FOG and resume normal gait. RAS might be ineffective due to the latency between the start of a FOG event, its detection and initialization of RAS. We propose a system capable of both FOG prediction and detection using signals from tri-axial accelerometer sensors that will be useful in initializing RAS with minimal latency. We compared the performance of several time frequency analysis techniques, including moving windows extracted from the signals, handcrafted features, Recurrence Plots (RP), Short Time Fourier Transform (STFT), Discreet Wavelet Transform (DWT) and Pseudo Wigner Ville Distribution (PWVD) with Deep Learning (DL) based Long Short Term Memory (LSTM) and Convolutional Neural Networks (CNN). We also propose three Ensemble Network Architectures that combine all the time frequency representations and DL architectures. Experimental results show that our ensemble architectures significantly improve the performance compared with existing techniques. We also present the results of applying our method trained on a publicly available dataset to data collected from patients using wearable sensors in collaboration with A.T. Still University.

## 1. Introduction

Parkinson’s Disease (PD) had affected about 6.2 million people globally in 2015 [1]. Since then the number is estimated to have risen to around 10 million [2], making it one of the most widely occurring neuro-degenerative movement disorders. PD is usually much more prevalent in aging people, adults 60 and over [3]. It is characterized by the loss of nerve cells or neurons in the substantia nigra area of the basal ganglia in the human brain. Loss of these cells results in reduction of the neurotransmitter dopamine and ultimately in decreased control of body movements [3,4,5,6].

PD is characterized by a number of neurological and motor symptoms like speech impediments, olfactory dysfunctions, autonomic dysfunctions, sleep disorders, fatigue, resting tremors, stiffness of trunk and limbs, slowness of movement (bradykinesia), reduced movement (akinesia), dyskinesia, irregular stride length and gait speed, Freezing of Gait (FOG), falls and postural disorders [7]. It is difficult to diagnose PD without the manifestation of motor symptoms, which are often unlikely to appear before 50% to 70% of the neurons have been damaged [8], making it difficult to administer any kind of preventive measures. The motor symptoms usually cause functional impairments in a subject, creating difficulties in sitting and standing up. The person with PD also suffers from losing the normal pendulum motion of the arms and displaying very small steps [4,5].

One of the most common symptoms of PD is FOG, with around 50% of all PD patients being affected [9,10]. Episodes of FOG cause the person with PD to intermittently experience a sudden inability to move, which often occurs while initiating gait, making turns while walking or when experiencing stress. The subjects report a feeling of their feet being glued to the ground during these events [11]. Based on the signals received from sensors worn around the ankles, it was found that while normal walking steps occur at a frequency of 0.5 Hz to 3 Hz, FOG exhibits a frequency of 6 Hz to 8 Hz [12,13].

Typically, FOG is very difficult to estimate and predict, but it can cause the risk of falls and pose health risk for the affected elderly [14,15,16,17]. An accurate prediction and detection of FOG can reduce accidents and thus improve the quality of life of the patients and their loved ones.

Current methods for detecting FOG [18,19,20] mostly consist of tests and detailed questionnaires used to assess the frequency and severity of FOG episodes. Although somewhat accurate, these methods suffer from shortcomings because of their clinical setup not reflecting real-world scenarios. FOG events usually tend to occur at home or while the patients are performing Activities of Daily Living (ADL) [7,11,21], which are different from the clinical test setup.

With the advancement in technology, using wearable sensors to monitor movements, body temperatures, heart rates and other physical parameters has become increasingly commonplace [22]. These sensors are lightweight, comfortable and usually do not hamper a person’s daily activities while monitoring ADL. The data recorded from wearable sensors for activity detection has brought promising performance in various applications, especially when combining with modern Machine Learning (ML) and Deep Learning (DL) based techniques [23,24,25,26,27]. There are wearable sensors that use auditory stimulation to treat FOG, which help shorten the duration of FOG events [28]. However, these sensors cannot effectively stop FOG episodes because of the latency of detection, which can still be hundreds of milliseconds in the best case scenarios [29].

There have been many applications using wearable technologies along with ML and DL based techniques to monitor motor functions of PD patients, aiming to achieve more effective treatment and reduce healthcare expenses [30,31,32]. These approaches can provide an unobtrusive and comfortable experience to the patient, while collecting personalized long term relevant medical history and improving the quality of treatment. Maetzler et al. [33] state that an automatic FOG analysis and detection system could play a vital role in monitoring the occurrence and evolution of FOG events over time. Although a permanent and guaranteed cure for PD or FOG itself has not been available at this time, a sufficiently accurate automatic monitoring system might prove to be helpful in minimizing the frequency and duration of FOG events. An established FOG treatment is to use Rhythmic Auditory Stimulation (RAS) [34], which produces a rhythmic ticking sound as auditory cues to help the patient resume normal gait when a FOG is detected. RAS has shown to improve walking by maintaining the speed and amplitude of movements [35,36,37].

With the popularity of the wearable sensors, the amount of available data collected from them is increasing at a rapid pace, which facilitates the use of DL based techniques. DL is under the scope of artificial intelligence that has the capabilities to automatically extract features from data without manual feature engineering. DL based end-to-end classifiers have shown promising performance, outperforming ML based classifiers in general, if sufficient amount of training data is available. Recently, DL based approaches have been adopted to perform tasks related to Human Activity Recognition (HAR) using data from various sensors [23,24,25,26].

Deep Convolutional Neural Networks (CNNs) are common Deep Learning architectures. Lecun et al. [38] mention in their book “The Handbook of Brain Theory and Neural Networks” that CNNs can be applied to temporal signals and images to automatically extract abstract distinct features by combining several convolutional operators. Although CNNs are proficient in extracting invariant local features from data, this architecture often falls short when the data has global time dependency, which is often the case with data obtained from wearable sensors. Recurrent Neural Networks (RNNs) are able to solve this issue because the connections between the nodes of this architecture exhibit a discrete-time dynamical system [39,40]. Long Short Term Memory (LSTM) is one of the most widely used RNNs, able to model time dependency in sequential time series data using various logic gates to control a memory space [41].

Neural Network Ensembling is the learning paradigm of training a collection of neural networks to collaborate on the same task [42]. The idea of ensembling was introduced by Hansen et al. [43], who proposed that the generalization ability of a Neural Network based system can be significantly improved by training a number of neural networks and by combining their solutions to solve the same problem. A typical ensemble architecture consists of two steps, i.e., training multiple components or constituent neural networks and then creating an architecture that combines their outputs. In recent years, Ensemble Learning techniques have been applied to PD detection tasks and they have achieved significant success [44,45].

The purpose of this work is to combine some most widely used time frequency analysis techniques, with CNN and LSTM based architectures for the detection and prediction of FOG events, using data captured from a tri-axial accelerometer sensor. In order to solve the issue of detection latency, we predict the changes in gait immediately before a FOG event. If the onset of FOG events can be accurately predicted, RAS can be applied even before it starts. We use a BiDirectional LSTM architecture with raw signals and handcrafted features and it was observed that handcrafted features did not improve the performance. We explored a CNN architecture with multiple visual representation methods including Recurrence Plot (RP), Short Time Fourier Transform (STFT), Discrete Wavelet Transform (DWT) and Pseudo Wigner Ville Distribution (PWVD). Three ensemble neural network architectures were also proposed and it was observed that they significantly improve the prediction and detection performance compared to individual models. We evaluated our models with multiple metrics to ensure that our findings are unbiased.

Our research group, in collaboration with Arizona School of Health Sciences, A.T. Still University, acquired gait data from 14 PD patients recorded using APDM^™^ wearable sensors. We applied our trained neural networks and proposed a system capable of monitoring FOG from these data after we verified the performance of our models on a publicly available dataset.

## 2. Literature Review

Smart sensors have been commonly used as a tool for assessing motor symptoms such as FOG in PD and other movement disorders. This is possible because of the improvements in computational power of small devices [46]. Existing FOG assessment methods using these sensors can be categorized into different groups depending on the sensor types, sensor locations, extracted features, and the analytics methods. FOG detection can be conducted real-time or offline [47]. However, FOG detection and prediction are challenging tasks because of the variability of event duration and frequency. We observe that previous studies mainly captured FOG episodes that are not consistent to the patients’ normal daily activities because their data were simulated in laboratory settings. In this section, we review related work on FOG detection.

An early FOG detection method was proposed by Han et al. [48] using U-AMS (Activity Monitoring System). Wavelet power features were used for discrimination of abnormal movements in PD patients. Moore et al. [49] then proposed a threshold based method for FOG detection by defining the Freeze Index (FI), which is the ratio between the power of the signal in “freeze” band (3–8 Hz) divided by the power of the signal in the “locomotion” band (0.5–3 Hz). The proposed method marks FOG episodes when FI exceeds a certain threshold. The subject dependent experimental results show 78% correct detection of FOG (true positive rate) and 20% false positive rate. Bachlin et al. [28] presented a real time FOG detection method by introducing a new term to Moore et al. [49] method, called Power Index (PI), which is the addition of walking band (WB) and Freezing Band (FB) that indicates the amount of movement. In [28], FOG episodes are determined using two thresholds (Freezing Threshold (FTH) and Power Threshold (PTH)) given FI>FTH and PI>PTH. In this method, once the FOG episodes are detected, the patient will get the auditory signals until his normal walking ability is resumed. They reported 73.1% and 81.6% for sensitivity and specificity respectively. The author also created the Daphnet dataset [28] for FOG assessment methods evaluation.

The first proposed FOG detection method based on ML was by Mazilu et al. [50]. The features for this classification were from the work of Bachlin et al. [28] with some additional features including mean, standard deviation, entropy, energy, FI and power of the acceleration signals. Random Forest (RF), Naive Bayes and K-nearest neighbour (KNN) were the ML algorithms used for classification. Motion data capture was done by a smartphone and a wrist acceleration sensor. The best obtained results were 66.25 and 95.83 for sensitivity and specificity respectively with RF using 10-fold cross-validation. In the following year, they presented another automatic FOG detection system using wearable sensor. In this work, they did multi-class analysis as the PreFOG motion was considered a new class (FOG vs. PreFOG vs. normal locomotion). Learning was conducted by studying the time domain and statistical features from the motion data. In this new work, they could improve F1 score by 8.1%. The new automatic FOG detection method introduced auditory cueing to warn the patient about FOG episodes. In the same year (2013), a system for automatic FOG detection was proposed by Tripoliti et al. [51]. The system was based on four steps: data imputation (interpolation), band-pass filtering, entropy calculation and automatic classification (Naïve Bayes, RF, Decision Trees and Random Tree). Data was obtained from 5 healthy subjects, 5 PD patients with FOG symptoms and 5 PD patients without FOG symptoms. The results show 81.94% sensitivity, 98.74% specificity, 96.11% accuracy and 98.6% Area Under Curve (AUC) using RF. Another proposed FOG detection work in 2013 was by Moore et al. [52], which assesses seven sensors placed in different locations for gait analysis. Their analysis found that the shank and back were the most convenient places for the sensors. However, they found that using all the seven sensors could get higher and more robust performance with sensitivity 84.3% and specificity 78.4%.

In 2015, Zack et al. [53] presented a threshold based FOG detection technique following the approach of Moore [52] using a single tri-axial accelerometer placed at the waist. Receiver operating characteristic (ROC) curves were drawn to determine a global FI threshold to distinguish between FOG and non-FOG episodes for different tasks. In addition to the global FI threshold, they calculated the sensitivity and specificity of the FI threshold for each subject. Combining all task results, a sensitivity of 75% and specificity of 76% were achieved [47].

Rodríguez et al. [54] presented a novel approach for FOG detection using machine learning techniques and daily activities of the PD patients in real environments. They extracted 55 FOG related features from 21 PD patients using just a single waist-worn tri-axial accelerometer. Support Vector Machine (SVM) with leave-one-out cross-validation was used for classification in two scenarios: user independent and user dependent. Experimental results show a sensitivity of 88.09% and specificity of 80.09% with R-10-fold cross-validation and a sensitivity of 79.03% and specificity of 74.67% for leave-one-subject-out (LOSO) evaluation. After that, Sama et al. [55] decreased the number of features to 28 for the same dataset. The extracted features were sent to 8 different classifiers with greedy subset selection process, 10-fold cross-validation and different window sizes. The results of FOG detection at patients’ homes were 91.7% and 87.4% for sensitivity and specificity respectively, which are better than the results of Rodrigues’s method.

Orphanidou et al. [56] evaluated machine learning algorithms to identify the FOG prior to its onset. An accelerometer time series dataset containing 237 individual Freezing of Gait events from 8 patients was considered, from which features were extracted and presented to 7 machine learning classifiers. SVM achieved the highest performance in comparison with the benchmark techniques. The classification algorithm was applied to 5 s windows using 18 features, obtaining balanced accuracies (the mean value of sensitivity and specificity) of 91%, 90% and 82% over the Walk, FOG and Transition classes, respectively. However, the need for systematic analysis of the problem was identified. Therefore, in their next study [56], they specifically focused on the early detection of a FOG event, through classification of the transition class using varying size time windows and time/frequency contrary to the majority of previous studies that recognized FOG only when it had occurred. In their paper, the Daphnet dataset was used with accelerometer signals obtained from sensors mounted on the ankle, thigh and trunk of the PD patients. Data augmentation was performed on the dataset to include another class label called ‘transition’ that showed the episodes before FOG occurrence. Daphnet features were sent out to a group of 5 classifiers, including Gradient Boosting (GB), Extreme Gradient Boosting, SVM, RF and Neural Networks. Experimental results show that SVM with Radial Basis (RBF) kernels has the best performance with sensitivity of 72.34%, 91.49%, 75.00% and specificity values of 87.36%, 88.51% and 93.62%, for FOG, transition and normal activity classes, respectively.

Deep Learning (DL) techniques have also been used for automatic FOG determination. DL can handle multi-modal data, missing information and high dimensional feature spaces. The first proposed FOG detection method using DL was by Camps et al. [57]. The proposed 1D Convolutions Neural Network (CNN) has 8 layers, which is trained using a novel spectral data representation strategy that considers information from both the previous and current signal windows. The data was collected from 21 subjects, consisting 9-channel signals recorded from a waist-worn Inertial Measurement Unit (IMU) with three tri-axial sensors: accelerometer, gyroscope and magnetometer. The experimental results show a performance of 90.6% for the Geometric Mean (GM), an AUC of 0.88, a sensitivity of 91.9% and a sensibility of 89.5%.

In 2019, San-Segundo et al. [58] presented a study to evaluate the robustness of different feature sets and ML algorithms for FOG detection using body-worn accelerometers. They used four feature sets: (Mazilu et al. [50] features, Human Activity Recognition (HAR) features, Mel Frequency Cepstral Coefficients (MFCCs) features, and Speech Quality Assessment (SQA) features). They also used four classifications (RF, multi-layer perceptron, Hidden Markov models (HMM) and deep neural networks). Evaluation was performed using a LOSO cross-validation. The best results were obtained when using the current window and three previous windows, with the feature set composed of Mazilu features [50] and MFCCs [59]. They found that the best classifier was a deep convolutions neural network achieving an AUC of 0.93 and an Equal Error Rate (EER) of 12.5%.

In 2020, Sigcha et al. [60] evaluated some ML and DL classification and detection techniques with accelerometer signals acquired from a body worn IMU to enhance the FOG detection performance in real-world home environments. Three data representations proposed in the literature were reproduced (including Mazilu features [50], Mel Frequency Cepstral Coefficients (MFCCs) [59] and Fast Fourier Transform (FFT)) to establish a baseline using RF classifier with 10-fold cross-validation (R10fold) and LOSO. This analysis was also conducted to find the best data representation to test DL approaches including: a denoiser autoencoder, a deep neural network with CNN and a combination of CNN and LSTM layers. For comparison purposes, shallow algorithms such as one-class SVM (OC-SVM), SVM, AdaBoost and RF were tested. This study was evaluated on the data collected by Rodríguez-Martín et al. [54], which includes recordings from 21 PD patients, who manifested FOG episodes when performing ADL at their homes. The best performance for AUC was 0.93. Their results illustrate that modeling spectral information of adjacent windows through an LSTM model can improve the performance of FOG detection without increasing the length of the analysis window.

In order to give a clear comparison of the different methods, we summarize their characteristics in Table 1. The novelty of the proposed work lies in the usage of multiple time frequency techniques as well as the usage of ensembling methodologies. Although there have been many experiments with ML and DL based technologies and feature extraction methods for FOG detection, to the best of our knowledge, our work is the first to apply time frequency representation techniques like Recurrence Plots (RP), Short Time Fourier Transform (STFT), Discreet Wavelet Transform (DWT) and Pseudo Wigner Ville Distribution (PWVD) alongside original signal and extracted features for FOG detection and prediction. We combine these techniques to design ensemble architectures. We designed a Bidirectional LSTM based architecture with four layers and trained separate instances of the architecture on Raw signal windows as well as 27 features extracted from the signal window.

We also designed a CNN architecture with four Convolutional layers, separate instances of which were trained on our visual features (RP, STFT, DWT, PWVD). Combining these models trained six different data modalities, we proposed our three ensemble architectures and evaluated their performance. The proposed models were able to both detect and predict FOG events while achieving the high scores in multiple evaluation criteria. The scores of the proposed ensemble methods either outperform or are very close to the scores of existing methods. Note that when solving a multiclass classification problem, i.e., in both identifying and predicting FOG events, our architectures achieve superior performance. We also tested the trained models on monitoring progression with real-world data, i.e., predicting FOG from accelerometer data captured using APDM wearable sensors. The result demonstrated that our approach is able to detect preFOG episodes in real world scenarios as presented in Section 5.

We believed there was room for improving the performance of existing models. None of the existing works analyzed by us had used visual representation of time series data for this task. Combining various visual representation techniques with handcrafted feature engineering and the capacity of DL based models to automatically extract relevant features from raw data was a promising avenue of research. Furthermore, although some authors including Orphanidou et al. [56] had introduced the concept of a PreFOG/Transition state before entering FOG state, their average performance scores for all classes were comparatively low and in their experiment SVM outperformed Neural Network based architectures. In other cases where DL based architectures achieved superior performance, they were mostly detecting FOG itself, not the PreFOG state. The goal of this experiment was to develop a unified solution that would be able to both predict FOG by detecting the PreFOG state and detect FOG itself with high accuracy. Additionally, we also wanted to compare the performance of various different time frequency representation techniques for this task.

## 3. Materials and Methods

In this work, we developed DL based techniques and used time frequency representation data as a feature set to classify as well as predict the FOG events. Experimental results show that our approach gives higher accuracy compared with existing state-of-the-art models based on tri-axial accelerometer sensor signals. The performance of each DL model was evaluated with different feature sets and multiple metrics in order to determine the optimal combination of models without bias. Finally, we are able to propose three ensemble architectures, each of which is composed of a selected set of models and features. The ensemble architectures significantly improve the performance of individual models.

### 3.1. Data

**Figure 1:**
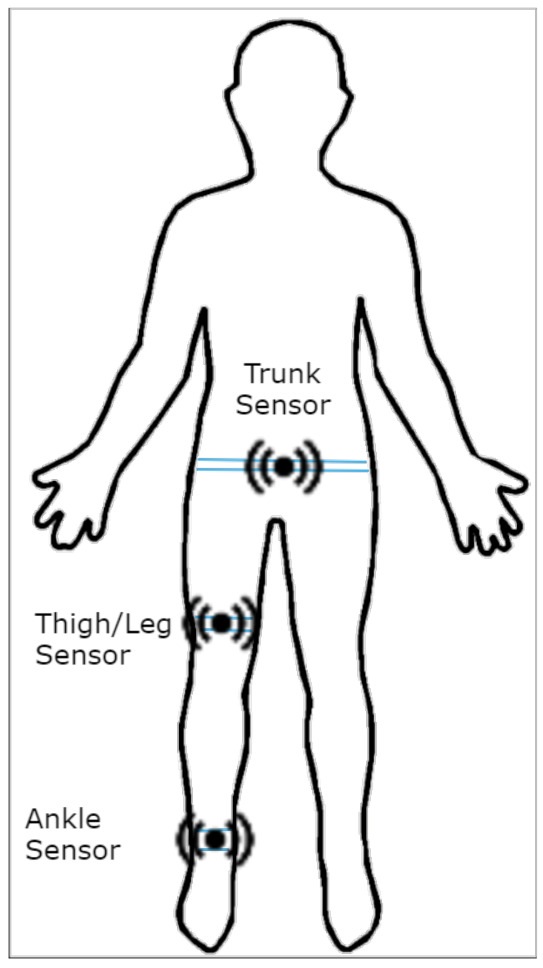
Sensor placement for data collection.

The publicly available DAPHNet [28] dataset was used for our experiments. The dataset contained data collected from ten PD patients, with seven male and three female experiencing regular FOG in their day to day activities. The average age of the participants was 66.4±4.8 years, with an average disease duration of 13.7±9.67 years. The average Hoehn and Yahr score was 2.6±0.65, indicating that the subjects had mild symptoms with mild balance impairment to moderate balance impairment [61]. Two tri-axial (3D) accelerometer sensors were attached to one of the patient’s legs: One was located at the shank just above the ankle and another was attached to the thigh slightly above the knee. A third sensor was placed at the lower back of the patient. The locations of the sensors are given in [28] and illustrated in Figure 1.

The dataset contained 237 FOG events. Synchronized video recordings were used by physiotherapists to identify the FOG events. The signal point, where the left-right steps stop alternating, is defined as the start of a FOG event. The point, where normal pattern resumed is defined as the end of the FOG event. Eight out of the ten subjects experienced FOG during the study, with the duration of FOG events ranging from 0.5 s to 40.5 s. The mean duration was 7.3±6.7 s, with 50% of the FOG episodes being shorter than 5.4 s and 93.2% being shorter than 20 s. The signals were annotated in three categories:

- 0—Not part of the experiment; user performed activities are unrelated to the experimental protocol while the sensors were installed.
- 1—Experiment; no FOG.
- 2—FOG.

### 3.2. Proposed Data Preparation Method

**Figure 2:**
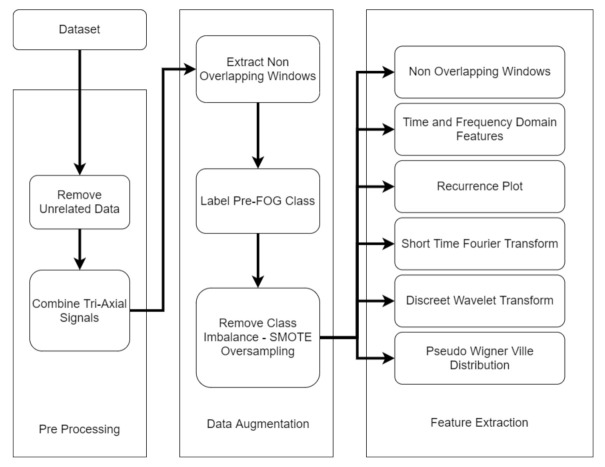
Proposed preprocessing, data augmentation and feature extraction workflow.

An overview of the preprocessing, data augmentation and feature extraction algorithms used in this study is presented in Figure 2. The major components are explained in the subsections below.

### 3.3. Preprocessing

In the preprocessing component, data that is irrelevant to the study is removed and signals from the three axes of each sensor is combined so that there is only one signal stream for each sensor.

#### 3.3.1. Removing Unrelated Data

As previously described in Section 3.1, data with an annotation of 0 was not a part of the experiment; the subjects performed activities which were not in accordance with the protocol of the data collection process. In this step, the unrelated data was excluded from the dataset.

The Daphnet [28] dataset was divided per subject and we excluded subjects who did not experience FOG at all during the data collection period.

#### 3.3.2. Calculating Magnitude of Acceleration for all three axis

Each of the accelerometers was assigned to a single channel, with the data being recorded for three channels: Ankle (A), Leg (L) and Torso (T) representing the locations of the sensor placement. Each channel contained three separate signals, with each of the signals corresponding to a single axis of the accelerometer. The axes were horizontal forward (X), vertical (Y) and horizontal lateral (Z). Thus, a set **τ** of nine signals was recorded for each of the patients with a sampling frequency ($f_{s}$) of 64, as illustrated in Equation (1).

(1) $$\tau =\{\left\{A_{X},A_{Y},A_{Z}\right\},\left\{L_{X},L_{Y},L_{Z}\right\},\left\{T_{X},T_{Y},T_{Z}\right\}\}$$

Magnitude of acceleration is the relative value of the overall acceleration at any given time instance, calculated as shown in Equation (2), combining the signals for each of three axes (x,y,z) into a signal.

(2) $$\alpha_{C}=\sqrt{\alpha_{X}^{2}+\alpha_{Y}^{2}+\alpha_{Z}^{2}},where\;\alpha_{X},\alpha_{Y},\alpha_{Z}\in \tau ,\;\alpha_{C}\in \tau_{C}$$

The three signals (x,y,z) originating from each channel (A,L,T) were combined to calculate the magnitude of acceleration denoted by $\tau_{C}$ in Equation (2), resulting in three signal streams with one from each of the sensors as shown in Equation (3).

(3) $$\tau_{C}=\left\{A_{C},L_{C},T_{C}\right\}$$

### 3.4. Data Augmentation

We applied small non-overlapping windows to extract data from the original continuous signal. The window data immediately before the start of a FOG event was labelled with a new class PreFOG, which is essential for predicting FOG events before they occur. The number of Non-FOG samples vastly outnumber the PreFOG and FOG samples, making Non-FOG our majority class. In order to solve the issue of class imbalance, the minority classes, PreFOG and FOG, were over sampled to match the number of samples from the Non-FOG class.

#### 3.4.1. Signal Segmentation

Non-overlapping 1-dimensional windows of length $f_{s}\times w$ time-steps were used to extract signal $\alpha_{C}\in \tau_{C}$, where *w* is the length of the signal window in seconds.

(4) $$\alpha_{C}={\left[\;\alpha_{1}\;\alpha_{2}\;\ldots \;\alpha_{w\times f_{s}}\;\right]}_{w\times f_{s}}$$

Since the windows were non-overlapping, shorter window lengths provided a larger dataset. Signals were segmented into window lengths ranging from 1 to 4 s.

#### 3.4.2. Labeling PreFOG class

Mazilu et al. [62] proposed that gait cannot enter into FOG state directly from normal walking without first going through a state of deterioration. They define this state as PreFOG, which is a transition period with variable duration. Identifying this transition state would be valuable for both FOG detection and prediction. Since the duration of PreFOG might not be the same from patient to patient, for our experiment the immediate window ($w\times f_{s}$ time steps) before the onset of a FOG event was labeled as PreFOG. The final dataset thus had three annotations,

- 0—Non FOG
- 1—FOG
- 2—Pre FOG

**Figure 3:**
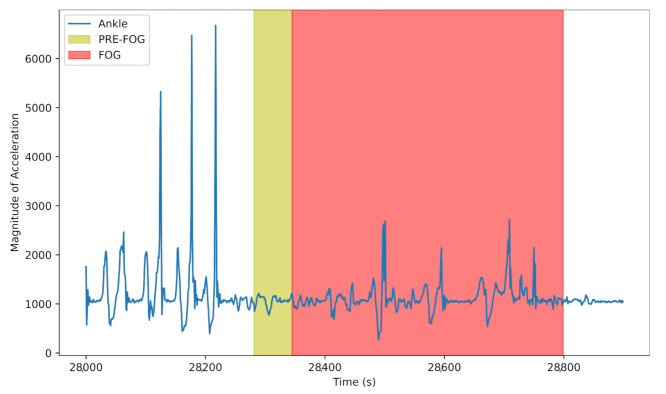
Example of combined Accelerometer signal from Ankle, capturing the motor variations in the gait of a PD patient, containing Normal gait, followed by a window of PreFOG period (Yellow), and then a FOG event (Red).

Figure 3 presents an illustration of combined accelerometer signal from Ankle that contains normal gait, PreFOG and FOG. PreFOG is highlighted in yellow and the FOG episode is highlighted in red.

#### 3.4.3. SMOTE Oversampling

At this stage, the dataset was hugely imbalanced, with the majority of the data being from the Non-FOG class. Such imbalanced data would lead to most architectures ignoring the minority classes and over-classifying the majority class, although the performance on the minority classes is much more significant in this case. There are multiple ways to address this issue. One approach is to under-sample the majority class to match the number of samples in the minority classes. However, in our case, the minority samples are sparse, and under-sampling the majority class would lead to a drastic decrease in the total number of training samples. Neural Network architectures require a large number of training samples in order to perform satisfactorily, and therefore under-sampling would lead to poor performance. An alternative method is to over-sample the minority class. It involves duplicating the samples of the minority class to match the number of samples in the majority class. Although this method balances the class distribution, it does not provide the networks with any new information to learn. We decided to choose the approach proposed by Chawla et al. [63] to synthesize new samples from existing samples. This Synthetic Minority Over-sampling Technique (SMOTE) creates new synthetic plausible samples that are in the same feature space as other minority class samples.

### 3.5. Feature Extraction

After data augmentation, our final feature set consisted of 5 different modalities extracted from the same source signal in addition to original signal itself, $\alpha_{i}\in \alpha_{C}$ as shown in Equation (5).

(5) $$Features_{i}=\left\{\alpha_{i},F_{i},RP_{i},STFT_{i},DWT_{i},PWVD_{i}\right\},\alpha_{i}\in \alpha_{C}$$

where:

- $\alpha_{i}$ = Moving window extracted from signal $\alpha_{C}$.
- $F_{i}$ = Manually extracted feature set from $\alpha_{i}$.
- $RP_{i}$ = Recurrence Plot representation of $\alpha_{i}$.
- $STFT_{i}$ = Short Time Fourier Transform representation of $\alpha_{i}$.
- $DWT_{i}$ = Discrete Wavelet Transform representation of $\alpha_{i}$.
- $PWVD_{i}$ = Pseudo Wigner Ville Distribution representation of $\alpha_{i}$.

#### 3.5.1. Time and Frequency Domain Features

For each moving window $\alpha_{i}\in \alpha_{C}$, 27 features relating to the time and frequency domain were extracted, as explained in Table 2. Some of the features were selected based on previous works done by Mazilu et al. [62] who added additional features to the work of Bachlin et al. [28] including Min, Max, Range, Median, Mode, Trimmed Mean, Standard Deviation, Variance, Root Mean Square, Skewness, Kurtisis, Normalized Signal Magnitude Area, Mean Crossing Rate and Signal Vector Magnitude. Additional time domain features such as Mean Absolute Value, Median Absolute Deviation, 25th and 75th percentile values, Interquantile Range and Peak of Fourier Transform were added. Furthermore, sensor and frequency based features such as Energy, Entropy, Peak Frequency, Freeze and Locomotion Band Power, Freeze Index and Band Power were added in accordance to the work done by Gokul et al. [64].

#### 3.5.2. Recurrence Plots

Recurrence Plots (RP) are used to represent temporal correlations of univariate series data defined in a square matrix [65]. For time series data, the matrix elements represent the times at which the amplitude of the signal recurs. If *i* and *j* are two time instances, and x(i) and x(j) are values in the time series at two recurrence time instances, the formula to compute the recurrence plot [66] is given in Equation (6)

(6) $$R\left(i,j\right)=\left\{\begin{array}{cc} 1, & if\;||x(i)-x(j)\leq \epsilon ||\\ 0, & \mathrm{otherwise} \end{array}\right.,\left(\epsilon \;is\;a\;custom\;similarity\;threshold\right)$$

**Figure 4:**
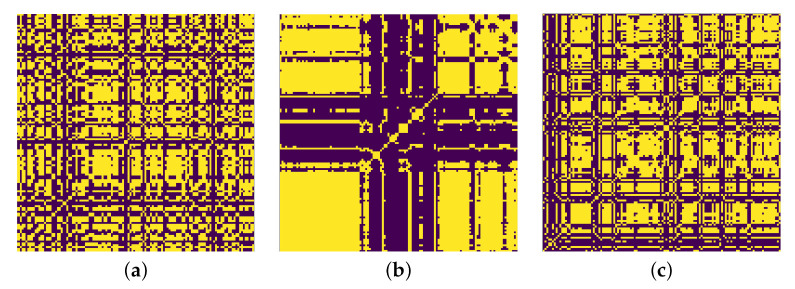
Examples of recurrence plot for (**a**) signals representing Normal walking or Non-FOG, (**b**) signals representing PreFOG and (**c**) signals representing FOG.

Recurrence plots are often robust against outliers and noisy data for periodic signals. Some examples of recurrence plots for our signals can be seen in Figure 4. The plots were generated with a window length (*w*) of 2. It was observed that for w=2, Non-FOG events had no distinct pattern when represented as a recurrence plot, PreFOG events show clear distinct patterns and FOG events had patterns that were more defined than Non-FOG but less defined than PreFOG. Both *x* and *y* axes represent time for RP.

#### 3.5.3. Short Time Fourier Transform

**Figure 5:**
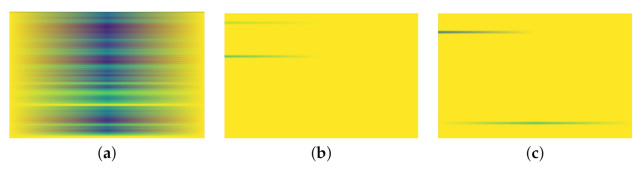
Examples of Short Time Fourier Transform plot for (**a**) signals representing Normal walking or Non-FOG, (**b**) signals representing PreFOG and (**c**) signals representing FOG.

A Short Time Fourier Transform (STFT) is a Fourier transform that quantifies the phase content and the sinusoidal frequency of signal segments changing over time [65]. STFT is useful in capturing the time and frequency characteristics in the signals. Rajoub et al. [67] mentioned that STFT does not perform well in capturing sharp signals and patterns with varying duration. Figure 5 shows some example spectograms generated using STFT, describing magnitude over time for each of our signal types over a 2 s time window. *x* axis represents time and *y* axis represents frequency for STFT.

For w=2, STFT captured the difference between Non-FOG and other classes, with the spectograms for PreFOG and FOG classes being almost clear compared to that of Non-FOG. However, it was difficult to visually differentiate PreFOG and FOG from STFT alone.

#### 3.5.4. Discrete Wavelet Transform

**Figure 6:**
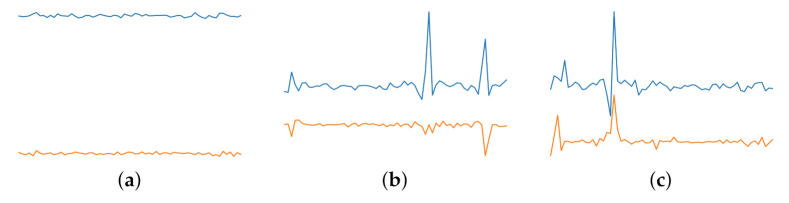
Discrete Wavelet Transformation plot for (**a**) signals representing Normal walking, (**b**) signals representing PreFOG and (**c**) signals representing FOG.

Discrete Wavelet Transform (DWT) is a process of decomposing a signal sequence into subsets, with each subset being a time series consisting coefficients that represent the time evolution in the corresponding frequency band [68]. A main advantage of DWT is the ability to capture both frequency and location characteristics in a time series. Haar Transform is the simplest of wavelet transforms. We used Haar sequence proposed by Haar et al. [69], which is the first known wavelet basis. The Haar wavelet can be used to analyze signals with sudden transitions, because of its non-differentiable property. Figure 6 shows sample plots of the approximation and detail coefficients of transforms for a 2 s time window, with 2 subsets. For DWT, *x* axis represents time and *y* axis frequency.

DWT representation plot for w=2 is useful for visually identifying the Non-FOG class compared to PreFOG and FOG classes. For Non-FOG events, the approximation and detail coefficient plots are almost flat, without any large fluctuation in value, which is distinctly identifiable. The representations for PreFOG and FOG events are harder to differentiate as both representations show sudden rise and drop in their values.

#### 3.5.5. Pseudo Wigner Ville Distribution

**Figure 7:**
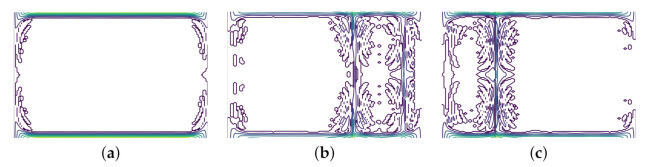
Examples of Pseudo Wigner Ville Distribution plot for (**a**) signals representing Normal walking, (**b**) signals representing PreFOG and (**c**) signals representing FOG.

Pseudo Wigner Ville Distribution (PWVD) is a method to represent transient phenomena in three dimensions, i.e., time, frequency and amplitude [70]. PWVD has been proven to be effective in generating accurate time frequency representation, since its frequency and time resolutions are determined by the resolution of the signals and not by the duration [70]. Figure 7 shows some examples of PWVD computed on signals with 2 s time window from our data. For PWVD, *x* and *y* axes represent time and frequency respectively.

The Non-FOG and FOG gaits can be clearly distinguished from PWVD representations for w=2, as Non-FOG gaits have a clear central section compared to FOG events. Both PreFOG and FOG classes have patterns appearing in the central section, which makes it difficult to differentiate them visually.

Finally, we can conclude that since RP shows a clear distinction between Normal and PreFOG states, and STFT, DWT and PWVD show a clear distinction between Normal and FOG states, if we combine RP with any one of STFT, DWT or PWVD, it can be possible to visually identify Normal, FOG or PreFOG states. This can be utilized to identify the resumption of Normal Gait after an FOG event.

### 3.6. Model Structure

Based on the findings obtained from the four feature visual representations, RP, STFT, DWT, PWVD discussed above, we introduced a Convolutional Neural Network (CNN) based model architecture. A Long Short Term Memory (LSTM) based architecture was proposed for the original signal α and the corresponding feature set *F*. For each data modality ∈Features, an instance of the corresponding model was trained and its performance was recorded. Then, the trained model instances were combined in three ensemble network architectures, M7, M8 and M9, as explained below. Our objective is to demonstrate that properly designed ensemble models can provide better performance than individual constituent models.

#### 3.6.1. Basic Convolutional Neural Network

**Figure 8:**
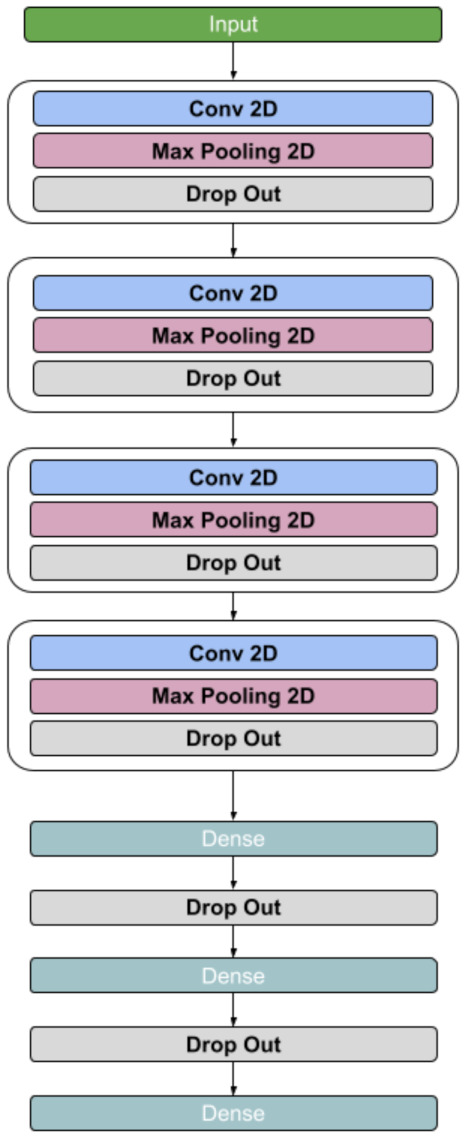
Proposed basic CNN Architecture with 4 recurring 2D Convolution blocks, followed by 3 Dense layers.

Convolutional Neural Networks (CNNs) are known for their ability to identify complex non-linear relationships between data points without hand crafted feature engineering. To complement our techniques to present time series data visually, a CNN architecture was designed, which is presented in Figure 8. The input is passed through four 2D Convolutional layers with filter sizes 64, 32, 16 and 8 respectively, a kernel size of (4,4) and LeakyReLu activation function with a negative slope coefficient and alpha value of 0.3. Each of the Convolutional layers was followed by a 2D MaxPooling layer with a pool size of (2,2) and a Dropout layer having a dropout rate of 0.25. The data was then flattened and passed through two Dense layers with 100 and 50 units respectively. Each of the Dense layers had LeakyReLu activation function with alpha value of 0.3 and was followed by 2 Dropout layers having a dropout rate of 0.2. Finally, a Dense layer with Softmax activation function of 3 units for our three output classes was added. The model was compiled with a RMSProp optimizer with an initial Learning rate of 0.0001. For our four visual feature types, RP, STFT, DWT and WV, a separate instance model was trained and validated, which are labeled M1,M2,M3 and M4 respectively.

#### 3.6.2. Basic Bidirectional LSTM

**Figure 9:**
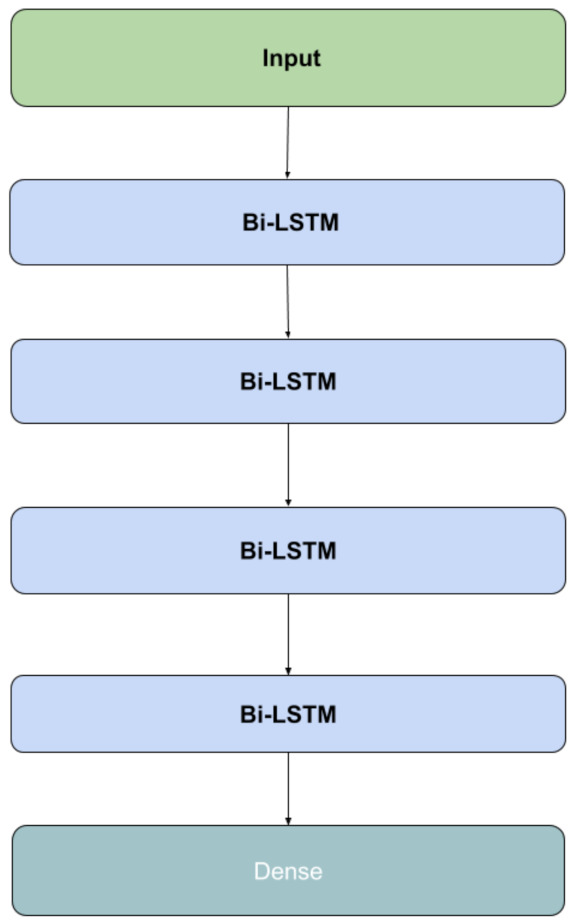
Proposed basic Bidirectional LSTM Architecture with 4 recurring Bidirectional LSTM blocks, followed by a Dense Layer.

Long Short Term Memory (LSTM) network is a type of recurrent neural network architecture, which is suitable for learning and remembering a long sequence of input data, automatically extracting features from the raw sequence and providing comparable performance to using handcrafted features. Bidirectional LSTMs add a duplication of the first recurrent layer. The first layer is trained on the original input sequence and the duplicated layer is trained on a reversed copy of the input sequence. For our data, the use of Bidirectional LSTM is justified because the context of the whole signal sequence, instead of a linear interpretation, is relevant for FOG identification and prediction. Our Bidirectional LSTM architecture is illustrated in Figure 9. The input is passed through four Bidirectional LSTM layers stacked on top of each other with tanh activation function and $n_{layers}$ hidden layers. The value of $n_{layers}$ is computed by Equation (7) where $l_{input}$ is the length of the input sequence and σ is the multiplication coefficient. The value for σ was set to 3 based on trial and error as it generated the best result. The output of LSTM was passed through a Dense layer with Softmax activation function. The final Dense layer had 3 units to classify between the three output classes. An Adam optimizer with an initial learning rate of 0.0001 was used to compile the model. One instance of this model, M5, was trained on the original signal $\alpha_{C}$ and another instance, M6, was trained on the handcrafted feature set $F_{C}$ corresponding to the signal $\alpha_{C}$.

(7) $$n_{layers}=l_{input}\times \sigma$$

#### 3.6.3. Ensemble Architectures

**Figure 10:**
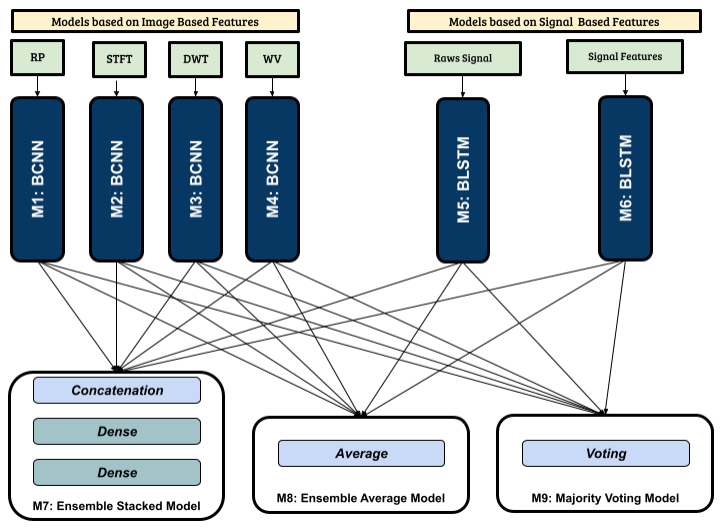
Proposed Ensemble Architectures, (1) M7 concatenates the output all constituent models, followed by a Dense Layer, (2) M8 Averages the outputs of all constituent models and (3) M9 calculates the majority prediction of all models using mode.

Ensemble Learning is a neural network training approach, where the predictions from multiple trained networks are combined to solve a problem [71]. In this work, three ensemble network architectures are examined (Figure 10). The constituent model set is defined as,

(8) $$M_{constituent}=\left\{M1,M2,M3,M4,M5,M6\right\}$$

##### Stacked Ensemble Model—M7

This model architecture is designed by combining the output predictions of all $M_{i}\in M_{constituent}$. The models have already been trained on their respective data, and all layers of constituent models are set as non-trainable before adding them to the ensemble model. The outputs of the models are passed through a Concatenation layer and then two Dense layers with 10 and 3 units respectively. The first Dense layer has a ReLu activation function and the final Dense layer has a Softmax activation function. An Adam optimizer is used with a learning rate of 0.0001.

##### Average Ensemble Model—M8

This model architecture takes the average of the predicted outputs of all $M_{i}\in M_{constituent}$. The constituent models pre-trained on their respective data are set as non trainable, and the outputs are passed through an Average layer. M8 is compiled with an Adam optimizer having a learning rate of 0.0001.

##### Majority Voting—M9

For majority voting, the output is based on the majority vote of the constituent models $M_{i}\in M_{constituent}$. The hard voting approach is used to calculate the final outcome, where every constituent model votes for an output class and the majority vote is selected as the final prediction. In statistical terms, this is equivalent to calculating the Mode of the predictions from all constituent models.

## 4. Results

The focus of this work is to analyze how well our proposed solution can detect and predict the characteristics embedded in the given data. We use collected data, which already has ground truth defined, i.e., True Positive (TP), and therefore do not need to obtain user opinions to validate the outcome. Instead, we compare the model generated results with the ground truth. We follow the assessment metrics based on TP, False Positive (FP), True Negative (TN) and False Negative (FN), used in related work in order to make fair comparisons.

### 4.1. Evaluation Criteria

In cases, where a majority class dominates the dataset, it might be possible that the detection accuracy is very high despite the model failing to identify the minority classes. To ensure that the performance of our model is properly evaluated and it is not over classifying the majority class, a number of evaluation metrics were utilized in our study.

#### 4.1.1. Detection Accuracy

Detection accuracy is the most widely used evaluation metric. It is defined as the fraction of predictions by a model that are accurate. Detection accuracy can be computed as in Equation (9). The output of this metric ranges between (0,1), with 0 being completely inaccurate and 1 representing perfect prediction.

(9) $$Accuracy=\frac{Number\;\;of\;\;correct\;\;predictions}{Total\;\;number\;\;of\;\;records}$$

#### 4.1.2. Precision, Recall/Sensitivity, Specificity, $F_{\beta }$ Score

Precision, Recall/Sensitivity, Specificity and $F_{\beta }$ Score are very important in understanding the performance of a model. Since we are evaluating a multi-class classification model, each of these metrics computes an individual class and then their weighted average is calculated.

Precision for a class is the measure of the classifier’s ability to not classify a negative sample as positive, as defined in Equation (10).

(10) $$Precision\left(A_{k},B_{k}\right)=\frac{|A_{k}\cap B_{k}|}{|A_{k}|}$$

Recall/Sensitivity of a class measures how well the classifier can identify positive samples of a class, as defined in Equation (11).

(11) $$Recall\left(A_{k},B_{k}\right)=\frac{|A_{k}\cap B_{k}|}{|B_{k}|}$$

Specificity for a class is defined as the ability of a classifier to reject samples that are not a member of that class.

(12) $$Specificity\left(C_{k},D_{k}\right)=\frac{|C_{k}\cap D_{k}|}{|D_{k}|}$$

The $F_{\beta }$ score is calculated as the weighted harmonic mean of Precision and Recall, ranging between (0,1), with 1 being the best possible value, as presented in Equation (13).

(13) $$F_{\beta }\left(A_{k},B_{k}\right)=\left(1+\beta^{2}\right)\frac{Precision\left(A_{k},B_{k}\right)\times Recall\left(A_{k},B_{k}\right)}{\beta^{2}Precision\left(A_{k},B_{k}\right)+Recall\left(A_{k},B_{k}\right)}$$

where,

- $A_{k}$ is the predictions for class *k*.
- $B_{k}$ is the occurrences for class *k*.
- $C_{k}$ is the predictions for samples not in class *k*.
- $D_{k}$ is the occurrences for samples not in class *k*.
- *k* represents a class in range 1:K, in our case K=3.

#### 4.1.3. Matthews Correlation Coefficient

Matthews Correlation Coefficient (MCC), also known as Phi Coefficient, was proposed by Matthews et al. [72] in 1975. MCC offers a balanced measure of quality for both binary and multi-class classifications, which can be used even if the classes are imbalanced. The value of this metric ranges from (−1,+1). A MCC value of +1 indicates perfect prediction, 0 indicates random prediction and −1 indicates inverse predictions. Gorodkin et al. [73] generalized MCC for multiple classes as the $R_{K}$ statistic, defined with respect to confusion matrix C for K classes following Equation (14) presented below [74].

(14) $$MCC=\frac{c\times s-\sum_{k}^{K}p_{k}\times t_{k}}{\sqrt{\left(s^{2}-\sum_{k}^{K}p_{k}^{2}\right)\times \left(s^{2}-\sum_{k}^{K}t_{k}^{2}\right)}}$$

where,

- $t_{k}=\sum_{i}^{K}C_{ik}$, the number of occurrences of class *k*.
- $p_{k}=\sum_{i}^{K}C_{Ki}$, the number of predictions for class *k*.
- $c=\sum_{k}^{K}c_{kk}$, total correct predictions.
- $s=\sum_{i}^{K}\sum_{j}^{K}Cij$, total number of samples.

### 4.2. K-Fold Cross Validation

In order to get an accurate estimate of the model performances, Stratified K-fold cross validation technique was utilized to separate the data into training and testing sets, with 80% of the data being used for training and the rest for testing, preserving the ratio of samples of each class. Since neural network models take a long time to train and evaluate, it is difficult to use high values for K. For this experiment, K was set to 5. The dataset was first shuffled, and then it was split into K unique (train, test) combinations. For each fold, a new instance of each of our models was trained using the training set and its performance on the testing set was evaluated and recorded. The evaluation performances were retained while the instances of the models were discarded. Finally, the average performance of the models across all K folds was recorded.

### 4.3. Normalization

The visual feature representations in $RP_{i},STFT_{i},DWT_{i},PWVD_{i}$ are normalized using Equation (15). Since the range for RGB values in images is (0–255), each channel is normalized to the range of 0–1. Then the values are centered through division with the mean.

(15) $$p_{normalized}=\frac{p_{o}-255.0}{mean(p_{o})},p_{o}\in RP_{i},STFT_{i},DWT_{i},PWVD_{i}$$

### 4.4. Experimental Setup

For each of the training sets, it was further divided into (train, validation) sets with 80% being used for training and the rest for validation. Validation using unseen data was crucial to evaluate whether the model was learning over time by comparing its performances. Each of the models was trained for 500 epochs. The training was stopped if the validation accuracy did not improve over 50 epochs. All the experiments were done on a Machine with Ubuntu OS, 4 core Intel Xeon Processor, 62 Gigabytes of RAM and Nvidia Tesla GPU with 16 Gigabytes memory. The algorithm was tested with data from all three wearable sensors $\alpha_{C}\in \tau_{C}$, but we achieved the best performance with data from the ankle mounted sensor $A_{C}$. All results presented here are based on $A_{C}$. In order to train Convolutional Neural Networks, all image features were adjusted to the shape of (3, 128, 128) and then normalized. The runtimes presented include both training and testing of the model but do not include the preprocessing and feature extraction time. Runtimes presented here for ensemble models do not include the time for training the constituent models. All scores presented in Section 4.5 are average scores. Five instances of each model were trained and evaluated on five Folds of (train, test) sets, and their scores and runtimes were averaged. Table 3 shows the reported performance of some existing models on the same dataset, in a (0–100) range.

### 4.5. Metric Scores and Discussion

Since we used non-overlapping time windows, smaller window sizes yielded significantly larger amount of data, which led to better performance in neural network based architectures. We experimented with widow sizes of 2, 3, 4 s. A window size of *w* seconds means that our model is able to predict the start of a FOG event *w* seconds before it happens. We believe that a window size of 1 would lead to much better detection performance since it means more training examples for the model. However, we did not use a smaller window size of 1 because it would also decrease the time window by which we can predict the FOG event, leading to a higher resource consumption during training.

We also observed that the size to which the features in $RP_{i}$, $STFT_{i}$, $DWT_{i}$, $PWVD_{i}$ are reshaped also plays a vital role in model performance, with larger sizes producing better results. Due to resource constraints, we set this size to be (3,128,128).

Section 4.5 presents the performance of for each model $M\in M_{1},\ldots M_{9}$ with signal $S\in \tau_{C}$ (for each of $Ankle(A_{C})$, $Leg(L_{C})$ and $Trunk(T_{C})$) with Window Size w∈2,3,4 (seconds). The tables report multiple evaluation metric scores including Accuracy, Precision, Recall/Sensitivity, Specificity, $F_{\beta }$ score, MCC score and the Runtime taken for the model to train in minutes. All scores are reported in the range of (0, 1), except MCC score, which is in the range of (−1, 1). The scores are reported in Mean±StandardDeviation format. The best scores for each model using the same modality of data but with different window sizes were reported in bold font.

From the metric scores presented in Table 4, it can be seen that Basic CNN M1 trained on RP generated from signals performs reasonably well across all metric scores. In most cases the smallest window size of 2 s yielded the best scores, but there was not a drastic decrease in performance when we increased the window sizes. Comparing sensor locations, data collected from Trunk sensor ($T_{C}$) performed the best, followed closely by data collected from Ankle ($A_{C}$) and Leg ($L_{C}$).

Table 5 presents the scores for Basic CNN M2 trained on STFT plots generated from the signals. For STFT. window size of 3 seemed to provide comparatively better results, although the scores were poor when compared to the scores from RP. The data collected from Trunk sensor ($T_{C}$) provided best results when using STFT, followed closely by data collected from Leg ($L_{C}$) and Ankle ($A_{C}$).

The metric scores of Basic CNN M3 using DWT are reported in Table 6. M3 achieved the highest accuracy among our models using visual features (RP, STFT, DWT, PWVD). The scores for varying window sizes were very similar, with a window size of 2 s providing the best scores for Ankle ($A_{C}$) and Trunk ($T_{C}$) sensor data. For data collected from the Leg ($L_{C}$), a window size of 3 generated the best scores. Comparing the scores of the three sensors’ locations, it was noted that Trunk ($T_{C}$) provided the best scores, followed very closely by Leg ($L_{C}$) and Ankle ($A_{C}$).

Table 7 notes the metric scores of Basic CNN M4 using PWVD. A window size of 3 s provided the best scores for Ankle ($A_{C}$) and Leg ($L_{C}$) sensor data. For data collected from the Trunk ($T_{C}$), a window size of 4 generated the best scores. Comparing the best scores for each sensor location, the scores for all three locations were pretty similar.

Table 8 contains the scores of Bidirectional LSTM with extracted raw signal windows. The overall performance is not as good as using visual features. The performance does not experience a drastic change when the window size increases from 2 to 3 s, but we see a significant drop in performance as the window size changes from 3 to 4 s. A window size of 3 s provided comparatively better scores for Trunk ($T_{C}$) and Leg ($L_{C}$) sensor data. For data collected from the Ankle ($A_{C}$), a window size of 2 generated the best scores. Data from Leg ($L_{C}$) sensor provided the best overall scores when using bidirectional LSTM and raw signals.

The scores of Bidirectional LSTM with extracted features are presented in Table 9. The performance is slightly better than using raw signals. The performance does not experience drastic changes with changes in the window size. A window size of 2 s provided comparatively better scores for Ankle ($A_{C}$), Trunk ($T_{C}$) and Leg ($L_{C}$) sensor data. Data from Ankle ($A_{C}$) sensor provided the best overall score. The results when using Trunk ($T_{C}$) sensor data slightly outperformed the scores when using Leg ($L_{C}$) sensor data.

The scores from LSTMs (M5 and M6) were moderate, but the issue was the very long runtime. The time for training LSTMs on raw signals was almost 5 times and features was almost 3 times of that for training the CNNs on visual features. All three of our ensemble architectures M7, M8 and M9, improved the scores of individual models. The majority voting model M9 had the best performance across all evaluation criteria without any extra training or parameter tuning. The scores were high for all evaluation criteria. The reported runtimes for ensemble models do not include the training time needed to prepare the constituent models.

Table 10 contains the scores of ensemble architecture M7 with all features. The overall performance is vastly superior to using individual features. The performance does not change significantly when the window size changes. A window size of 3 s generated the best scores for Ankle ($A_{C}$) and Leg ($L_{C}$) sensor data and a window size of 2 generated the best scores for data collected from the Trunk ($T_{C}$). Data from Leg ($L_{C}$) sensor provided the best overall scores. We can see that for M7, the runtimes were very large with all window sizes, which is a disadvantage considering this does not include the training time for individual models that make up the ensemble architecture. Adding the runtimes for constituent models, a significant amount of time was needed to train this model architecture.

Table 11 reports the scores of ensemble architecture M8 with all features. This architecture is similar to M7, except it adds an Average layer and calculates the average of the prediction of all constituent models, whereas M7 concatenates the predictions and uses two Dense layers to reshape the output. The overall performance is superior to using individual features and comparable to the performance of M7. The performance does not change significantly when the window size changes. For this model, window size of 3 s generated the best scores using Ankle ($A_{C}$) and Leg ($L_{C}$) sensor data and a window size of 2 generated the best scores for data collected from the Trunk ($T_{C}$). Data from Trunk ($T_{C}$) sensor provided the best overall scores. The runtimes for M8 were not that large when compared to M7, but the performances were comparable. Thus, adding the runtimes for constituent models, M8 was able to produce similar results while needing a lot less time for training.

Table 12 presents the scores of Majority voting architecture M9 with all features. This architecture is different from M7 and M8; there is no training for this method. The overall performance is similar to the performance of M7 and M8. The performance is not much affected when the window size changes. For this model, window size of 3 s generated the best scores using Ankle ($A_{C}$) sensor data and a window size of 2 generated the best scores for data collected from the Trunk ($T_{C}$) and Leg ($L_{C}$). Data from Ankle ($A_{C}$) sensor provided the best overall scores, very closely followed by Trunk ($T_{C}$) and Leg ($L_{C}$). However, the main strength of this model lies with its runtime, as it only outputs the majority result of its constituent models. Since there is no training time, it can generate the output in milliseconds. It can produce scores similar to M7 and M8 while not needing any extra training time.

The results were also compared with the performance of some state-of-the-art models on the same dataset, as shown in Table 3. Mazilu et al. [62] compared feature learning approaches based on time and statistical domain with unsupervised learning approaches using principle component analysis for both FOG detection and prediction. Their average sensitivity, specificity and $F_{\beta }$ score are presented for only the FOG class with both supervised and unsupervised approaches. Our proposed approach in this work outperforms their results for the FOG class. Moreover, the result they presented is only for the FOG class; their method had lower scores when identifying the PreFOG class.

Gokul et al. [64] presented a number of ML based techniques to detect FOG events and evaluate their performances with sensitivity and specificity, which are also presented in Table 3. They also experimented with multiple window sizes and achieved the best results with a window length of 4 s. Although their performance is higher than our proposed model, they solved a binary classification problem of only identifying the FOG event. Their work does not have a prediction component. They achieved the best results with Random Forest classifier, with a sensitivity score of 98.91 and a specificity score of 99.44. Our ensemble architecture results were very close to their scores, while being able to also predict the onset of a FOG event. However, compared to Gokul et al. [64], one shortcoming of our method would be the large size of the trained models, which might pose a problem in deploying the models to wearable sensors.

## 5. Application of Trained Model on Data Collected from APDM^™^ Sensors

We were able to obtain an additional dataset in collaboration with the Arizona School of Health Sciences, A.T. Still University. There were 14 PD patients in their experiments where gait was monitored and sensor data was recorded using APDM^™^ wearable sensors. Eight of the subjects were male and six were female. The subjects were aged between 55 and 77 years and the mean age of the subjects was 64.7. The average Hoenn & Yahr score for the subjects was 2.2 which indicated mild symptoms. The data made available to us was anonymous. Using the existing models trained on Daphnet [28] data and the Majority Voting Ensemble Architecture, our goal is to detect the onset of FOG in this real-world dataset.

### 5.1. Data Collection and Processing

The data was provided by the Arizona School of Health Sciences, A.T. Still University. The study was supervised, with the prior knowledge that 7 out of the 14 patients were Freezers, identified as a score > 0 on the New Freezing of Gait Questionnaire (NFOGQ) and the rest being non-Freezers, with a score of zero on the NFOGQ [20]. There were two different configurations of the Six-Minute Walk Test (6MWT), a common clinical assessment of walking endurance [75]: 50 and 100 feet. For both configurations, the duration of the study was fixed at 6 min. Each patient had to walk for 6 min continuously with a 180 degree turn after either a 50 or 100 feet walk respectively. The number of turns was less for the 100 feet configuration. For each of the 14 patients, two datasets were generated with one for each configuration. The data was cleaned and missing values were filled with zeros. Twenty-eight data files were generated with 14 containing Freezer data and 14 containing non-Freezer data.

#### Sensor Types and Locations

**Figure 11:**
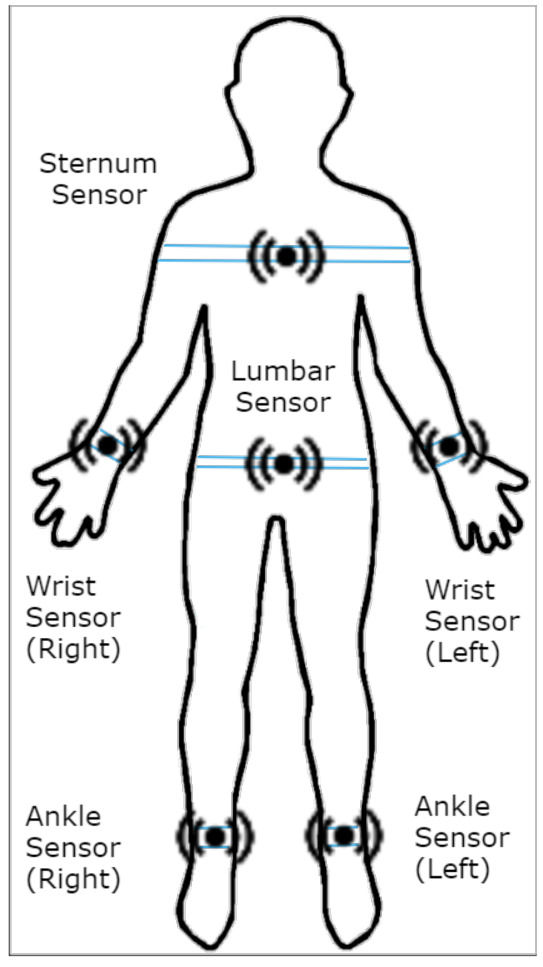
Sensor placement for data collection.

The sensors were placed in six locations on a patient’s body as illustrated in Figure 11.

The sensors were placed in Left Foot (Ankle), Right Foot (Ankle), Left Wrist, Right Wrist, Sternum and Lumber (Trunk).

For each location, the following five types of sensor were used to record data simultaneously:

- Accelerometer.
- Magnetometer.
- Gyroscope.
- Barometer.
- Temperature.

The recorded data were processed using Moveo Explorer^™^ and Mobility Lab^™^.

### 5.2. Workflow and Challenges

**Figure 12:**
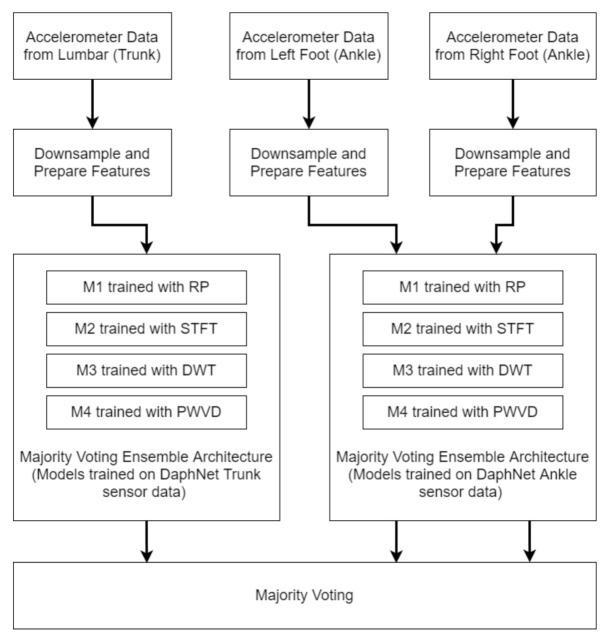
Workflow of monitoring FOG using models trained on Daphnet data.

Figure 12 depicts our workflow for this part of the experiment. In order to make the data consistent across both Moveo Explorer and Mobility Lab, MinMax normalization was used. Since our purpose was only to monitor FOG, we discarded all the data where the patient did not experience FOG. As the result, data of 7 out of the 14 patients was discarded.

Since there were multiple sensors in multiple locations, finding the appropriate sensors suitable for our experiment was an initial step. There are multiple sensors producing a large amount of data and processing them all can be both time and resource intensive. Furthermore, it could lead to biased predictions or impacting the performance negatively. Identifying the optimal combination of sensors which was the most useful in detecting FOG posed a challenge. We decided to consider only sensor locations that overlap with Daphnet [28] sensor locations, i.e., Left and Right foot (Ankle) and Lumber (Trunk). Although data from multiple sensors as well as derived kinematic information like velocities and displacements was available, we only considered accelerations. This was because our training dataset, Daphnet [28], only provided acceleration data, and thus it was not possible for our currently pretrained architecture to use data from additional sensors like Magnetometer, Gyroscope or Barometer. Our training dataset also did not consist of data from sensors placed on the wrist or sternum area of the patient. Therefore, signals from those areas would not be helpful in this case. We combined the signals from all three axis (X,Y,Z) using Equation (2).

The sampling frequency for the data was 128 Hz, which is double the sampling frequency of Daphnet [28]. Since our models were trained on a sampling frequency of 64 Hz, the data needed to be down-sampled from 128 Hz to 64 Hz in order for the trained models to be effective.

Although the data was labeled, the labels were for the whole time series. That means we used one continuous 6 min time series, which had only one label indicating whether FOG occurred in that series or not. However, the exact occurrence and number of FOG were unmarked; we did not know when exactly the FOG event happened or how many FOG events are there. FOG events usually last shorter than 1 min, so there might be multiple occurrences FOG within one 6 min signal. Thus even if we did apply our models trained on Daphnet [28] to this data, it would be difficult to verify the accuracy of the results. No significant difference can be visually defined between the two types of signal for this dataset, because the very short FOG episodes occur in between 6 min of regular walking. So even the majority of Freezer data is basically similar to the non-Freezer data, with some FOG episodes in between.

In order to detect the occurrence of even a small FOG, a window size of 2 was selected. After selecting the appropriate sensors and down-sampling the data, non-overlapping moving windows of 2 s were extracted from the source signal. Then the relevant features were generated from the windows. We only chose to use visual features RP,STFT,DWT,PWVD, since they have demonstrated good performance and were less time consuming as explained in the previous part of our experiment using the Daphnet [28] dataset.

After generating the features, the models M1, M2, M3 and M4 trained on Daphnet [28] data were used to analyze the data. There were three instances of trained models. For data from Left and Right foot, the instances of models trained on data from Ankle sensor of Daphnet was used. For data from Lumbar, models trained on data from Trunk sensor of Daphnet was used. Our training dataset Daphnet does not specify whether the sensors were located at the left or right side of the body. So Left and Right foot data was used with models trained on Ankle sensor. Finally, the results for each of the models were passed through a modified majority voting model M9, which generated the result.

**Figure 13:**
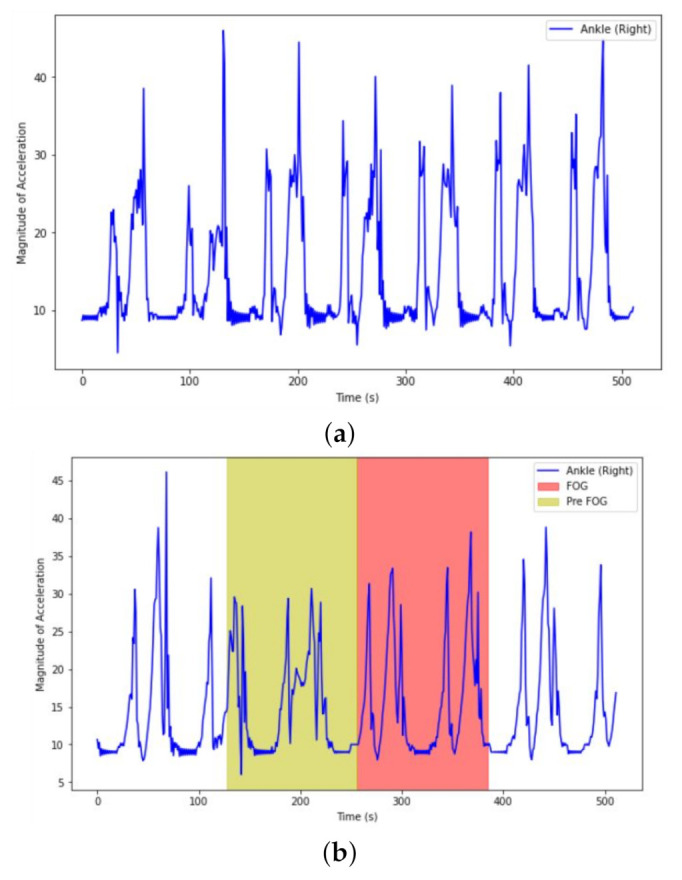
Comparison of Freezer vs. non-Freezer Accelerometer signals with (**a**) non-Freezer and (**b**) Detected preFOG and FOG from the Right Leg (Ankle).

For comparison, Figure 13 shows a small four window chunk of the signals from Accelerometer sensor of the right Ankle with a window size of 2 s. Since our data was down-sampled, this signal sequence has a sampling rate of 64 Hz instead of 128 Hz. The first Figure 13a shows a part of the sequence that does not contain any FOG or preFOG according to our model. Figure 13b shows a 4 s where our model identified PreFOG and FOG occurrences marked Yellow and Red respectively. When we compare the two figures, we can visually identify some differences. For the preFOG region, all the peak heights are smaller than that of non-FOG signals. The FOG region has higher peaks than that of preFOG, but the average peak height still appears to be lower than that of non-FOG signals. These patterns appear to be consistent with our observations from the Daphnet [28] data presented in Figure 3.

## 6. Conclusions

In this work, the performance of multiple time frequency representation techniques was compared in detecting and predicting FOG using tri-axial accelerometer sensor data from the publicly available Daphnet [28] dataset. We presented three ensemble neural network architectures comprised of multiple modalities of data and analyzed their performances. We were able to validate that ensemble network architectures significantly improve the performance over individual models.

The novelty of this work lies in the usage of multiple time frequency techniques and the usage of ensembling methodologies. Although there have been many experiments with ML and DL based technologies and feature extraction methods for FOG detection, as far as our results indicate and the literature review shows, this work is the first to apply time frequency representation techniques, i.e., Recurrence Plots, Short Time Fourier Transform, Discreet Wavelet Transform and Pseudo Wigner Ville Distribution, alongside captured signals and extracted features for FOG detection and prediction. Based on these innovative ideas, we have introduced effective ensemble architectures. Furthermore, the proposed solution is able to both detect and predict FOG events while achieving high performance scores based on multiple evaluation criteria. Our proposed ensemble methods outperform or are competitive to existing methods as demonstrated in Table 3, Table 4, Table 5, Table 6, Table 7, Table 8, Table 9, Table 10, Table 11 and Table 12. Mazilu et al. [50] reported a sensitivity and specificity score of 66.25% and 95.23% respectively. Tripoliti et al. [51] achieved 81.94% sensitivity, 98.74% specificity and 96.11% detection accuracy. Sama et al. [55] used eight different classifiers achieving 91.7% and 87.4% for sensitivity and specificity respectively. Gokul et al. [64] report the best scores with Random Forest (89.91% Sensitivity and 99.44% Specificity), AdaBoost (97.99% specificity and 99.56% sensitivity), SVM (97.54% Sensitivity and 98.64% Specificity) and ProtoNN (95.25% Sensitivity and 99.66% Specificity) using a window size of 4 to solve a binary problem of FOG detection only. Comparatively, using a window size of 3, our Majority Voting Architecture M9 achieved accuracy, sensitivity and specificity of 98.5%, 98.5% and 97.9% solving a multiclass classification problem of both FOG detection and prediction. When we used a larger window size of 4, the sensitivity and specificity were around 96.9% and 96.7%. We also evaluated our models using MCC scores (i.e., 1 is the best), and the scores of our ensemble architectures were always above 0.9 which indicates that our approach had excellent performance in all confusion matrix categories for all classes. While solving a multiclass classification problem, i.e., in both identifying and predicting FOG events, our architectures achieve superior performance across all evaluation criteria.

We also applied the trained models to monitor the progression of real-world data, i.e., FOG from accelerometer data captured using APDM^™^ wearable sensors, which demonstrate that our approach is able to detect preFOG episodes in real world scenarios. In future works, we will integrate more sensors and data modalities, investigate more model combinations for creating ensemble architectures, reduce size and complexity of the models and finally apply the resultant models to more real-world data. More importantly, we will extend our models in identifying the preFOG class, i.e., predicting FOG events, to use real-world data from more practical wearable sensors, in order to test the potential of preventing falls by initiating RAS even before the start of the event. One of the drawbacks of the proposed system is that the performance was not verified when applied on some collected data due to the lack of proper annotations. Thus, our future goals also include properly testing the performance of our method with more precisely annotated data using wearable sensors. The ultimate objective is to deliver a system that is capable of administering RAS for the prediction of FOG and the prevention of falls.

## Figures and Tables

**Table 1:** Comparison between technologies of various approaches.

Authors	Technology Used
Han et al. [48]	U-AMS (Activity Monitoring System) using wavelet power features for discrimination of abnormal movement.
Moore et al. [49]	Threshold based method for FOG detection by defining the Freeze Index (FI), achieving 78% True Positive Rate
Bachlin et al. [28]	Added Power Index (PI) to the method proposed by Moore et al. [49]
Bachlin et al. [28]	Two thresholds (FTH and PTH) given FI>FTH and PI>PTH, achieving 73.1% sensitivity and 81.6% specificity
Mazilu et al. [50]	Additional Features along with Bachlin et al. [28] features used with ML models RF, Naive Bayes and KNN, achieving 66.25% sensitivity and 95.23% specificity
Tripoliti et al. [51]	Data imputation (interpolation), band-pass filtering, entropy calculation, and automatic classification (Naïve Bayes, RF, Decision Trees and Random Tree), resulting in 81.94% sensitivity, 98.74% specificity, 96.11% accuracy and 98.6% AUC
Moore et al. [52]	Assessed seven sensors placed in different locations and concluded that the shank and back were the most convenient places for the sensors. Using all the seven sensors achieved best performance with sensitivity 84.3% and specificity 78.4%
Zack et al. [53]	presented a threshold based FOG detection technique following Moore [52] using ROC to determine a global FI threshold to distinguish between FOG and non-FOG episodes for different tasks, achieving sensitivity of 75% and specificity of 76%
Rodríguez et al. [54]	used ML techniques and 55 FOG related features from 21 PD patients with SVM. reporting a sensitivity of 88.09% and specificity of 80.09% for user independent evaluation, and a sensitivity of 79.03% and specificity of 74.67% for LOSO evaluation
Sama et al. [55]	Decreased the number of features to 28 for the Rodríguez et al. [54] dataset and used 8 different classifiers with greedy subset selection process, 10-fold cross-validation and different window sizes, achieving 91.7% and 87.4% for sensitivity and specificity respectively
Orphanidou et al. [56]	Extracted features and presented to 7 machine learning classifiers, with SVM achieving the highest performance. The classification algorithm was applied to 5 s windows using 18 features, obtaining balanced accuracy (the mean value of sensitivity and specificity) of 91%, 90%, and 82% over the Walk, FOG and Transition classes, respectively
Orphanidou et al. [56]	Focused on the early detection, through classification of the transition class using varying size time windows and time/frequency contrary to the majority of previous studies that recognized FOG only when it had occurred by including another class label called ‘transition’ that showed the episodes before FOG occurrence. 5 classifiers were used, including Gradient Boosting (GB), Extreme Gradient Boosting, SVM, RF and Neural Networks. SVM with RBF kernels has the best performance with sensitivity of 72.34%, 91.49%, 75.00% and specificity values of 87.36%, 88.51% and 93.62%, for FOG, transition and normal activity classes, respectively
Camps et al. [57]	Proposed 1D Convolutions Neural Network (CNN) with 8 layers, trained using a novel spectral data representation strategy that considers information from both the previous and current signal windows showing a performance of 90.6% for the GM, an AUC of 0.88, a sensitivity of 91.9% and a sensibility of 89.5%
San-Segundo et al. [58]	Evaluated the robustness of different feature sets and ML algorithms for FOG detection using body-worn accelerometers with 4 feature sets: (Mazilu et al. [50], HAR, MFCCs, and SQA) using 4 classification methods (RF, multi-layer perceptron, HMM and deep neural networks).The current window and three previous windows, with the feature set composed of Mazilu features [50] and MFCCs [59] achieved best scores. Deep convolutions neural network achieved an AUC of 0.93 and an Equal Error Rate (EER) of 12.5%.
Sigcha et al. [60]	Reproduced Mazilu features [50], MFCCs [59], and FFT) to establish a baseline using RF classifier with 10-fold cross-validation (R10fold) and LOSO. Further, tested multiple DL approaches include: A denoiser autoencoder, a deep neural network with CNN and a combination of CNN and LSTM layers and compared with shallow algorithms such as one-class SVM (OC-SVM), SVM, AdaBoost and RF, achieving 0.93 AUC
Our Proposed Method	Using all three sensors from Daphnet [28] dataset, used moving windows extracted from the original signal, handcrafted feature set and time frequency visualization techniques including RP, STFT, DWT and PWVD alongside a CNN and LSTM architecture. Evaluated the performance and used trained models to create 3 ensemble architectures which further improve the performance. The proposed method is capable of both FOG detection and prediction and the evaluation scores show better performance when compared to existing approaches.

**Table 2:** $F_{i}$ Features extracted for each $\alpha_{i}\in \alpha_{C}$.

Time Domain Features	Description
Min, Max	Minimum and Maximum value of the signal
Range	Difference between the minimum and maximum value of the signal
Mean	Average value of signal
Median	Median value of the signal
Mode	Modal value of the signal
Trimmed Mean	Trimmed/Truncated mean of the signal
Standard Deviation	Deviation of a signal compared to its mean
Variance	Square root of the standard deviation of the signal
Root mean square	Square root of the mean of the squared signal
Mean absolute value	Mean of absolute value of the signal
Median absolute deviation	Median over the absolute deviations from the median
25th Percentile	25th percentile value of the signal
75th Percentile	75th percentile value of the signal
Interquantile range	Difference between the 75th and 25th percentile of the signal
Normalized Signal Magnitude Area	Sum of standardized acceleration magnitude normalized by window length
Skewness	The degree of asymmetry in the signal
Kurtosis	The degree of peakedness in the signal, signals with high kurtosis have more outliers
Mean Crossing Rate	The number of times the signals goes from above average value to below average value normalized by the window length
Signal Vector Magnitude	Sum of euclidean norm over the window normalized by window length
Peak of Fourier Transform	Maximum magnitude of Discrete Fourier Transform of the signal normalized by the window length
**Frequency Domain Features**	**Description**
Entropy	Measure of random distribution of frequency
Energy	Sum of squared magnitude of FFT of the signal divided by window length
Peak Frequency	Maximum frequency value in the power spectrum
Freeze Band Power	The sum of power in Freeze band of frequencies divided by sampling frequency
Locomotion Band Power	The sum of power in Locomotion band of frequencies divided by sampling frequency
Freeze Index	Power of signal in freeze band (3-8Hz) divided by its Power in locomotion band(0.5-3Hz)
Band Power	Sum of the power in freeze band and in locomotion band

**Table 3:** The Metric scores of some related works on similar data.

Model	Window (s)	Sensitivity	Specificity	$F_{\beta }$ Score
Mazilu et al. [62] (Unsupervised—20 Features)	3	76.86	86.21	81.56
Mazilu et al. [62] (Supervised—20 Features)	3	66.65	88.74	78.27
Mazilu et al. [62] (Unsupervised—25 Features)	3	76.86	85.52	80.82
Mazilu et al. [62] (Supervised—25 Features)	3	67.58	88.52	78.65
Decision Tree [64]	4	96.70	98.92	-
Random Forest [64]	4	98.91	99.44	-
AdaBoost [64]	4	97.99	99.56	-
KNN [64]	4	94.61	97.38	-
SVM [64]	4	97.54	98.64	-
ProtoNN [64]	4	95.25	99.66	-
Bonsai [64]	4	92.9	98.36	-

**Table 4:** Results of Basic CNN M1 with RP.

Data Type	Window (s)	Accuracy	Precision	Sensitivity	Specificity	$F_{\beta }$ Score	MCC	Runtime (min)
$A_{C}$ (3)	2	**0.894 ± 0.021**	**0.929 ± 0.006**	**0.894 ± 0.021**	**0.933 ± 0.007**	**0.904 ± 0.016**	0.687 ± 0.039	52.05
	3	0.832 ± 0.023	0.905 ± 0.011	0.832 ± 0.023	0.926 ± 0.010	0.849 ± 0.021	0.648 ± 0.035	17:41
	4	0.864 ± 0.030	0.901 ± 0.020	0.864 ± 0.030	0.925 ± 0.020	0.873 ± 0.027	**0.695 ± 0.058**	**11:52**
$L_{C}$ (3)	2	**0.891 ± 0.019**	**0.929 ± 0.008**	**0.891 ± 0.019**	**0.937 ± 0.008**	**0.902 ± 0.01**6	0.684 ± 0.037	57:31
	3	0.873 ± 0.005	0.915 ± 0.006	0.873 ± 0.005	0.937 ± 0.006	0.883 ± 0.005	**0.702 ± 0.014**	22:41
	4	0.840 ± 0.022	0.895 ± 0.013	0.840 ± 0.022	0.930 ± 0.014	0.853 ± 0.020	0.664 ± 0.037	**9:25**
$T_{C}$ (3)	2	**0.926 ± 0.013**	**0.942 ± 0.007**	**0.926 ± 0.013**	**0.944 ± 0.009**	**0.930 ± 0.011**	**0.755 ± 0.033**	64:58
	3	0.876 ± 0.021	0.921 ± 0.008	0.876 ± 0.021	0.924 ± 0.008	0.889 ± 0.017	0.649 ± 0.038	15:24
	4	0.849 ± 0.000	0.911 ± 0.000	0.849 ± 0.000	0.934 ± 0.000	0.863 ± 0.000	0.667 ± 0.000	**9:59**

**Table 5:** Results of Basic CNN M2 with STFT.

Data Type	Window (s)	Accuracy	Precision	Sensitivity	Specificity	$F_{\beta }$ Score	MCC	Runtime (min)
$A_{C}$ (3)	2	0.678 ± 0.016	**0.893 ± 0.008**	0.678 ± 0.0176	0.860 ± 0.007	0.742 ± 0.011	0.407 ± 0.014	68.11
	3	**0.799 ± 0.030**	0.883 ± 0.010	**0.799 ± 0.030**	0.897 ± 0.012	**0.819 ± 0.026**	0.585 ± 0.038	18:41
	4	0.781 ± 0.030	0.877 ± 0.005	0.781 ± 0.030	**0.905 ± 0.01**0	0.803 ± 0.025	**0.588 ± 0.026**	**11:22**
$L_{C}$ (3)	2	0.719 ± 0.023	0.889 ± 0.009	0.719 ± 0.023	0.864 ± 0.017	0.762 ± 0.019	0.452 ± 0.035	127:59
	3	**0.815 ± 0.025**	**0.902 ± 0.006**	**0.815 ± 0.025**	**0.921 ± 0.008**	**0.834 ± 0.021**	**0.633 ± 0.030**	21:02
	4	0.746 ± 0.036	0.865 ± 0.010	0.746 ± 0.036	0.894 ± 0.013	0.770 ± 0.031	0.550 ± 0.032	**8:55**
$T_{C}$ (3)	2	0.781 ± 0.010	0.899 ± 0.002	0.781 ± 0.010	0.894 ± 0.003	0.813 ± 0.008	0.516 ± 0.008	57:19
	3	**0.831 ± 0.037**	**0.911 ± 0.013**	**0.831 ± 0.037**	0.914 ± 0.019	**0.854 ± 0.030**	0.586 ± 0.060	14:36
	4	0.816 ± 0.005	0.905 ± 0.004	0.816 ± 0.005	**0.927 ± 0.003**	0.836 ± 0.004	**0.629 ± 0.004**	**8:19**

**Table 6:** Results of Basic CNN M3 with DWT.

Data Type	Window (s)	Accuracy	Precision	Sensitivity	Specificity	$F_{\beta }$ Score	MCC	Runtime (min)
$A_{C}$ (3)	2	**0.939 ± 0.002**	**0.948 ± 0.004**	**0.939 ± 0.002**	0.947 ± 0.009	**0.942 ± 0.002**	0.785 ± 0.013	67.44
	3	0.922 ± 0.031	0.940 ± 0.019	0.922 ± 0.031	**0.949 ± 0.017**	0.926 ± 0.028	**0.796 ± 0.068**	22:39
	4	0.906 ± 0.022	0.923 ± 0.018	0.906 ± 0.022	0.941 ± 0.017	0.910 ± 0.021	0.766 ± 0.050	**13:48**
$L_{C}$ (3)	2	0.923 ± 0.005	0.941 ± 0.001	0.923 ± 0.005	0.944 ± 0.006	0.928 ± 0.004	0.748 ± 0.003	127:32
	3	**0.940 ± 0.010**	**0.951 ± 0.006**	**0.940 ± 0.010**	**0.963 ± 0.003**	**0.943 ± 0.009**	**0.834 ± 0.020**	26:43
	4	0.930 ± 0.020	0.940 ± 0.015	0.930 ± 0.020	0.958 ± 0.014	0.932 ± 0.019	0.817 ± 0.044	**16:47**
$T_{C}$ (3)	2	**0.946 ± 0.015**	**0.954 ± 0.010**	**0.946 ± 0.015**	0.952 ± 0.010	**0.949 ± 0.013**	0.811 ± 0.042	55:17
	3	0.938 ± 0.019	0.949 ± 0.012	0.938 ± 0.019	**0.952 ± 0.008**	0.941 ± 0.017	0.784 ± 0.048	22:12
	4	0.943 ± 0.008	0.953 ± 0.005	0.943 ± 0.008	0.971 ± 0.003	0.945 ± 0.008	**0.839 ± 0.019**	**12:39**

**Table 7:** Results of Basic CNN M4 with PWVD.

Data Type	Window (s)	Accuracy	Precision	Sensitivity	Specificity	$F_{\beta }$ Score	MCC	Runtime (min)
$A_{C}$ (3)	2	0.831 ± 0.023	0.906 ± 0.006	0.831 ± 0.023	0.902 ± 0.011	0.852 ± 0.018	0.571 ± 0.035	74.00
	3	**0.865 ± 0.015**	**0.907 ± 0.009**	**0.865 ± 0.015**	**0.930 ± 0.010**	**0.876 ± 0.014**	**0.681 ± 0.031**	32:34
	4	0.825 ± 0.024	0.888 ± 0.010	0.825 ± 0.024	0.919 ± 0.010	0.839 ± 0.021	0.641 ± 0.035	**17:46**
$L_{C}$ (3)	2	0.811 ± 0.011	0.901 ± 0.005	0.811 ± 0.011	0.897 ± 0.009	0.836 ± 0.009	0.545 ± 0.021	131:00
	3	**0.871 ± 0.020**	**0.912 ± 0.005**	**0.871 ± 0.020**	**0.930 ± 0.005**	**0.881 ± 0.016**	**0.695 ± 0.026**	28:58
	4	0.831 ± 0.025	0.895 ± 0.010	0.831 ± 0.025	0.923 ± 0.013	0.846 ± 0.021	0.657 ± 0.032	**16:27**
$T_{C}$ (3)	2	0.805 ± 0.011	0.900 ± 0.010	0.805 ± 0.011	0.887 ± 0.017	0.832 ± 0.010	0.531 ± 0.036	68:28
	3	0.842 ± 0.033	0.908 ± 0.009	0.842 ± 0.033	0.917 ± 0.013	0.861 ± 0.026	0.590 ± 0.052	29:24
	4	**0.864 ± 0.025**	**0.916 ± 0.010**	**0.864 ± 0.025**	**0.946 ± 0.012**	**0.877 ± 0.021**	**0.687 ± 0.040**	**15:50**

**Table 8:** Results of Bidirectional LSTM M5 with Raw Signals.

Data Type	Window (s)	Accuracy	Precision	Sensitivity	Specificity	$F_{\beta }$ Score	MCC	Runtime (min)
$A_{C}$ (3)	2	**0.797 ± 0.106**	0.896 ± 0.027	**0.797 ± 0.106**	0.917 ± 0.048	**0.827 ± 0.082**	0.527 ± 0.149	302.21
	3	0.784 ± 0.124	**0.903 ± 0.018**	0.784 ± 0.124	**0.939 ± 0.053**	0.817 ± 0.092	**0.597 ± 0.152**	149:31
	4	0.695 ± 0.178	0.832 ± 0.053	0.695 ± 0.178	0.774 ± 0.074	0.715 ± 0.155	0.441 ± 0.168	**73:22**
$L_{C}$ (3)	2	0.705 ± 0.106	0.877 ± 0.012	0.705 ± 0.106	0.836 ± 0.034	0.747 ± 0.088	0.418 ± 0.091	121:56
	3	**0.894 ± 0.021**	**0.929 ± 0.006**	**0.894 ± 0.021**	**0.933 ± 0.007**	**0.904 ± 0.016**	**0.687 ± 0.039**	96.09
	4	0.360 ± 0.380	0.315 ± 0.432	0.360 ± 0.380	0.806 ± 0.134	0.312 ± 0.419	0.246 ± 0.351	52:05
$T_{C}$ (3)	2	0.715 ± 0.270	0.880 ± 0.071	0.715 ± 0.270	0.800 ± 0.120	0.744 ± 0.239	0.478 ± 0.314	199:10
	3	**0.778 ± 0.142**	**0.903 ± 0.032**	**0.778 ± 0.142**	**0.930 ± 0.060**	**0.814 ± 0.111**	**0.531 ± 0.181**	105:22
	4	0.441 ± 0.312	0.517 ± 0.372	0.441 ± 0.312	0.728 ± 0.059	0.411 ± 0.305	0.111 ± 0.157	**74:20**

**Table 9:** Results of Bidirectional LSTM M6 with Extracted features.

Data Type	Window (s)	Accuracy	Precision	Sensitivity	Specificity	$F_{\beta }$ Score	MCC	Runtime (min)
$A_{C}$ (3)	2	**0.846 ± 0.034**	**0.912 ± 0.015**	**0.846 ± 0.034**	0.902 ± 0.023	**0.865 ± 0.028**	0.600 ± 0.067	179.36
	3	0.815 ± 0.020	0.896 ± 0.007	0.815 ± 0.020	**0.923 ± 0.009**	0.834 ± 0.017	**0.620 ± 0.026**	91:14
	4	0.803 ± 0.033	0.875 ± 0.010	0.803 ± 0.033	0.893 ± 0.009	0.821 ± 0.028	0.597 ± 0.040	**49:37**
$L_{C}$ (3)	2	**0.822 ± 0.013**	**0.907 ± 0.008**	**0.822 ± 0.013**	0.904 ± 0.012	**0.845 ± 0.010**	0.563 ± 0.025	147:41
	3	0.812 ± 0.011	0.897 ± 0.001	0.812 ± 0.011	**0.917 ± 0.002**	0.832 ± 0.008	**0.620 ± 0.011**	89:48
	4	0.783 ± 0.070	0.859 ± 0.042	0.783 ± 0.070	0.855 ± 0.043	0.801 ± 0.062	0.557 ± 0.122	**47:42**
$T_{C}$ (3)	2	**0.840 ± 0.007**	**0.907 ± 0.014**	**0.840 ± 0.007**	**0.898 ± 0.025**	**0.859 ± 0.006**	**0.579 ± 0.039**	190:06
	3	0.773 ± 0.022	0.900 ± 0.001	0.773 ± 0.022	0.897 ± 0.004	0.807 ± 0.017	0.519 ± 0.019	61:44
	4	0.770 ± 0.019	0.888 ± 0.013	0.770 ± 0.019	0.909 ± 0.019	0.797 ± 0.017	0.560 ± 0.035	**40:11**

**Table 10:** Results of Stacked Ensemble M7 with Extracted features.

Data Type	Window (s)	Accuracy	Precision	Sensitivity	Specificity	$F_{\beta }$ Score	MCC	Runtime (min)
$A_{C}$ (3)	2	0.971 ± 0.007	0.972 ± 0.007	0.971 ± 0.007	0.956 ± 0.012	0.971 ± 0.007	0.885 ± 0.027	200.32
	3	**0.979 ± 0.002**	**0.979 ± 0.002**	**0.979 ± 0.002**	**0.977 ± 0.005**	**0.979 ± 0.002**	**0.934 ± 0.006**	157:30
	4	0.967 ± 0.005	0.967 ± 0.007	0.967 ± 0.005	0.967 ± 0.012	0.967 ± 0.006	0.905 ± 0.018	**108:52**
$L_{C}$ (3)	2	0.967 ± 0.008	0.968 ± 0.008	0.967 ± 0.008	0.954 ± 0.013	0.967 ± 0.008	0.870 ± 0.032	200:29
	3	**0.980 ± 0.002**	**0.980 ± 0.002**	**0.980 ± 0.002**	**0.977 ± 0.005**	**0.980 ± 0.002**	**0.938 ± 0.006**	132:45
	4	0.965 ± 0.011	0.965 ± 0.011	0.965 ± 0.011	0.968 ± 0.014	0.965 ± 0.011	0.899 ± 0.032	**118:05**
$T_{C}$ (3)	2	**0.972 ± 0.009**	**0.973 ± 0.009**	**0.972 ± 0.009**	0.956 ± 0.013	**0.972 ± 0.009**	0.889 ± 0.036	324:54
	3	0.971 ± 0.006	0.971 ± 0.006	0.971 ± 0.006	**0.960 ± 0.008**	0.971 ± 0.006	0.882 ± 0.024	196:53
	4	0.967 ± 0.009	0.971 ± 0.007	0.967 ± 0.009	0.979 ± 0.003	0.968 ± 0.009	**0.900 ± 0.026**	**178:51**

**Table 11:** Results of Average Ensemble M8 with Extracted features.

Data Type	Window (s)	Accuracy	Precision	Sensitivity	Specificity	$F_{\beta }$ Score	MCC	Runtime (min)
$A_{C}$ (3)	2	0.979 ± 0.008	0.978 ± 0.009	0.979 ± 0.008	0.958 ± 0.013	0.978 ± 0.009	0.913 ± 0.034	103.09
	3	**0.980 ± 0.005**	**0.980 ± 0.005**	**0.980 ± 0.005**	**0.976 ± 0.007**	**0.980 ± 0.005**	**0.938 ± 0.015**	43:36
	4	0.967 ± 0.005	0.967 ± 0.006	0.967 ± 0.005	0.967 ± 0.012	0.967 ± 0.006	0.905 ± 0.018	**32:09**
$L_{C}$ (3)	2	0.973 ± 0.008	0.973 ± 0.008	0.973 ± 0.008	0.956 ± 0.013	0.973 ± 0.008	0.893 ± 0.032	107:14
	3	**0.978 ± 0.003**	**0.978 ± 0.002**	**0.978 ± 0.003**	**0.976 ± 0.003**	**0.978 ± 0.003**	**0.931 ± 0.008**	51:31
	4	0.969 ± 0.008	0.970 ± 0.008	0.969 ± 0.008	0.969 ± 0.013	0.969 ± 0.008	0.911 ± 0.024	**24:04**
$T_{C}$ (3)	2	**0.983 ± 0.006**	**0.983 ± 0.006**	**0.983 ± 0.006**	**0.960 ± 0.012**	**0.983 ± 0.006**	**0.932 ± 0.026**	64:56
	3	0.975 ± 0.005	0.975 ± 0.005	0.975 ± 0.005	0.962 ± 0.009	0.975 ± 0.005	0.900 ± 0.019	32:55
	4	0.976 ± 0.008	0.978 ± 0.007	0.976 ± 0.008	0.983 ± 0.003	0.976 ± 0.008	0.925 ± 0.024	**38:40**

**Table 12:** Results of Majority Voting M9 with Extracted features.

Data Type	Window (s)	Accuracy	Precision	Sensitivity	Specificity	$F_{\beta }$ Score	MCC	Runtime (min)
$A_{C}$ (3)	2	0.981 ± 0.007	0.980 ± 0.007	0.981 ± 0.007	0.951 ± 0.015	0.980 ± 0.007	0.921 ± 0.029	< 1
	3	**0.985 ± 0.003**	**0.985 ± 0.003**	**0.985 ± 0.003**	**0.979 ± 0.006**	**0.985 ± 0.003**	**0.953 ± 0.010**	< 1
	4	0.969 ± 0.006	0.969 ± 0.007	0.969 ± 0.006	0.967 ± 0.012	0.969 ± 0.007	0.911 ± 0.019	< 1
$L_{C}$ (3)	2	**0.977 ± 0.008**	**0.977 ± 0.008**	**0.977 ± 0.008**	0.958 ± 0.012	**0.977 ± 0.008**	0.907 ± 0.032	< 1
	3	0.973 ± 0.008	0.975 ± 0.006	0.973 ± 0.008	**0.974 ± 0.002**	0.973 ± 0.007	**0.917 ± 0.020**	< 1
	4	0.971 ± 0.008	0.972 ± 0.008	0.971 ± 0.008	0.967 ± 0.008	0.971 ± 0.008	0.917 ± 0.023	< 1
$T_{C}$ (3)	2	**0.983 ± 0.007**	**0.983 ± 0.007**	**0.983 ± 0.007**	0.960 ± 0.012	**0.983 ± 0.007**	**0.932 ± 0.030**	< 1
	3	0.977 ± 0.003	0.977 ± 0.004	0.977 ± 0.003	0.962 ± 0.008	0.976 ± 0.004	0.905 ± 0.015	< 1
	4	0.976 ± 0.011	0.978 ± 0.009	0.976 ± 0.011	**0.979 ± 0.004**	0.976 ± 0.010	0.925 ± 0.032	< 1

## Data Availability

The Daphnet dataset used for training the model is publicly available. Supplementary code will be made available on request to the correspondent author’s email with appropriate justification.

## Abbreviations

The following abbreviations are used in this manuscript:

FOG	Freezing of Gait
PD	Parkinson’s Disease
RAS	Rhythmic Auditory Stimulation
RP	Recurrence Plot
STFT	Short Time Fourier Transform
DWT	Discreet Wavelet Transform
PWVD	Pseudo Wigner Ville Distribution
DL	Deep Learning
ML	Machine Learning
LSTM	Long Short Term Memory
CNN	Concolutional Neural Network
RNN	Recurrent Neural Network
ADL	Activities of Daily Living
HAR	Human Activity Recognition
WB	Walking Band
FB	Freezing Band
FTH	Freezing Threshold
PTH	Power Threshold
FI	Freeze Index
PI	Power Index
RF	Random Forest
KNN	K Nearest Neighbours
AUC	Area Under Curve
ROC	Receiver Operating Characteristic
SVM	Support Vector Machine
LOSO	Leave One Subject Out
GB	Gradient Boosting
RBF	Radial Basis Function
IMU	Inertial Measurement Unit
SQA	Speech Quality Assesment
MFCC	Mel Frequency Cepstral Coefficients
EER	Equal Error Rate
FFT	Fast Fourier Transform
MCC	Matthews Correlation Coefficient
TP	True Positive
FP	False Positive
TN	True Negative
FN	False Negative
2AFC	Two-Alternative-Forced-Choice
DSCQS	Double Stimulus Continuous Quality Scale
U-AMS	Activity Monitoring System
GM	Geometric Mean
SQA	Speech Quality Assessment
HMM	Hidden Markov Model
